# Supplementary material for: End-to-end differentiable blind tip reconstruction for noisy atomic force microscopy images
Source: Sci Rep. 2023 Jan 4;13:129. doi: 10.1038/s41598-022-27057-2 (PMC9813222; doi:10.1038/s41598-022-27057-2)
Supplement: Supplementary file 1 — Supplementary Figures. [file 41598_2022_27057_MOESM1_ESM.pdf]

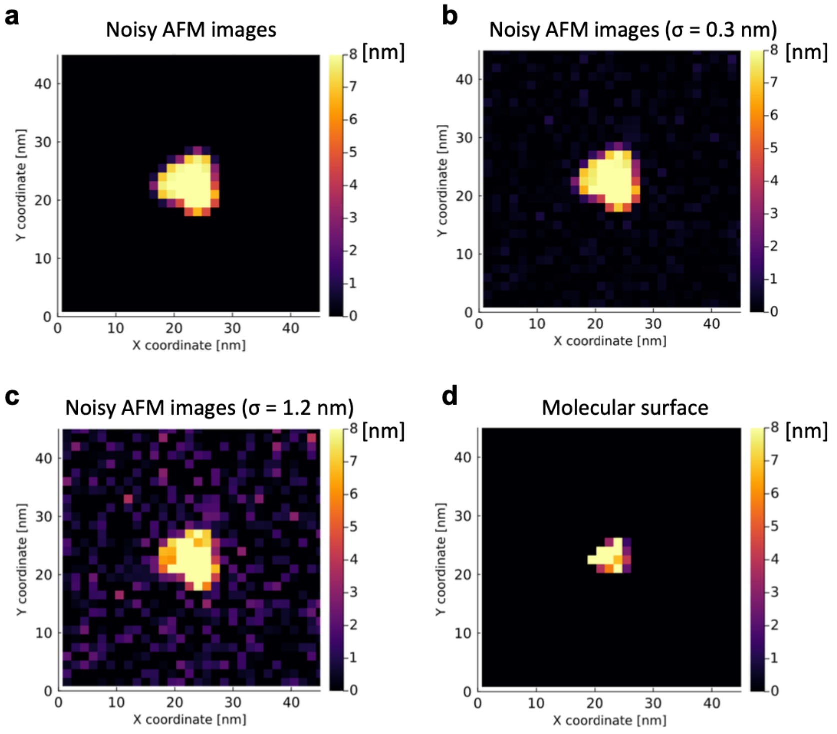

Supplementary Figure 1. Full-size images **a**, 1st frame of noise-free pseudo-AFM images. **b**, 1st frame of noisy pseudo-AFM images (with a standard deviation of $\sigma = 0.3$). **c**, 1st frame of noisy pseudo-AFM images (with a standard deviation of $\sigma = 1.2$). **d**, Molecular surface of the 1st frame of pseudo-AFM images.

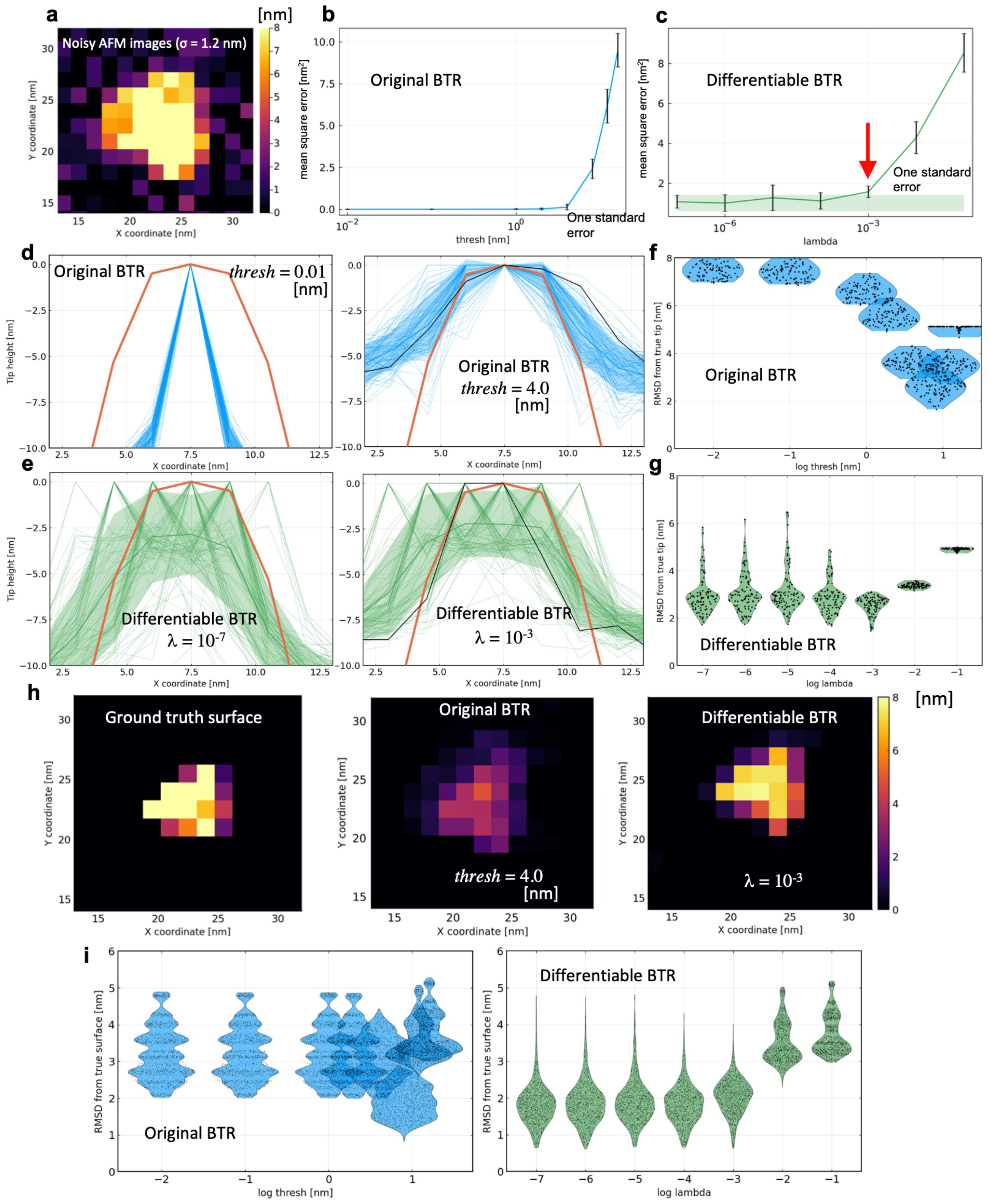

Supplementary Figure 2. Results of twin experiment using a large noise level with a standard deviation of $\sigma = 1.2$ nm. **a**, 1st frame of 20 images used for the twin experiment. **b-c**, Mean square errors (MSEs) optimized at various parameter values. The mean and standard deviation of MSE was calculated only from the single set of 20-frame images using the cross validation. Shaded area indicates the one standard error bound. The red arrow indicates the selected λ for deconvolution. **d-e**, Cross sections of reconstructed tip shapes along the x-axis with the original and differentiable blind tip reconstruction (indicated by dashed blue lines and dashed green lines, respectively), compared with the ground truth (red line). Shaded area represents the standard

deviation. The tip shapes used for deconvolution are indicated by the black lines. **f-g**, Root mean square deviations (RMSDs) of the reconstructed tips from the ground truth visualized by violin plots (with the same coloring scheme). **h**, Reconstructed molecular surfaces by the deconvolutions with the reconstructed tips. **i**, RMSDs of the deconvoluted molecular surfaces of all frames (20 images) from the ground truths visualized by violin plots.

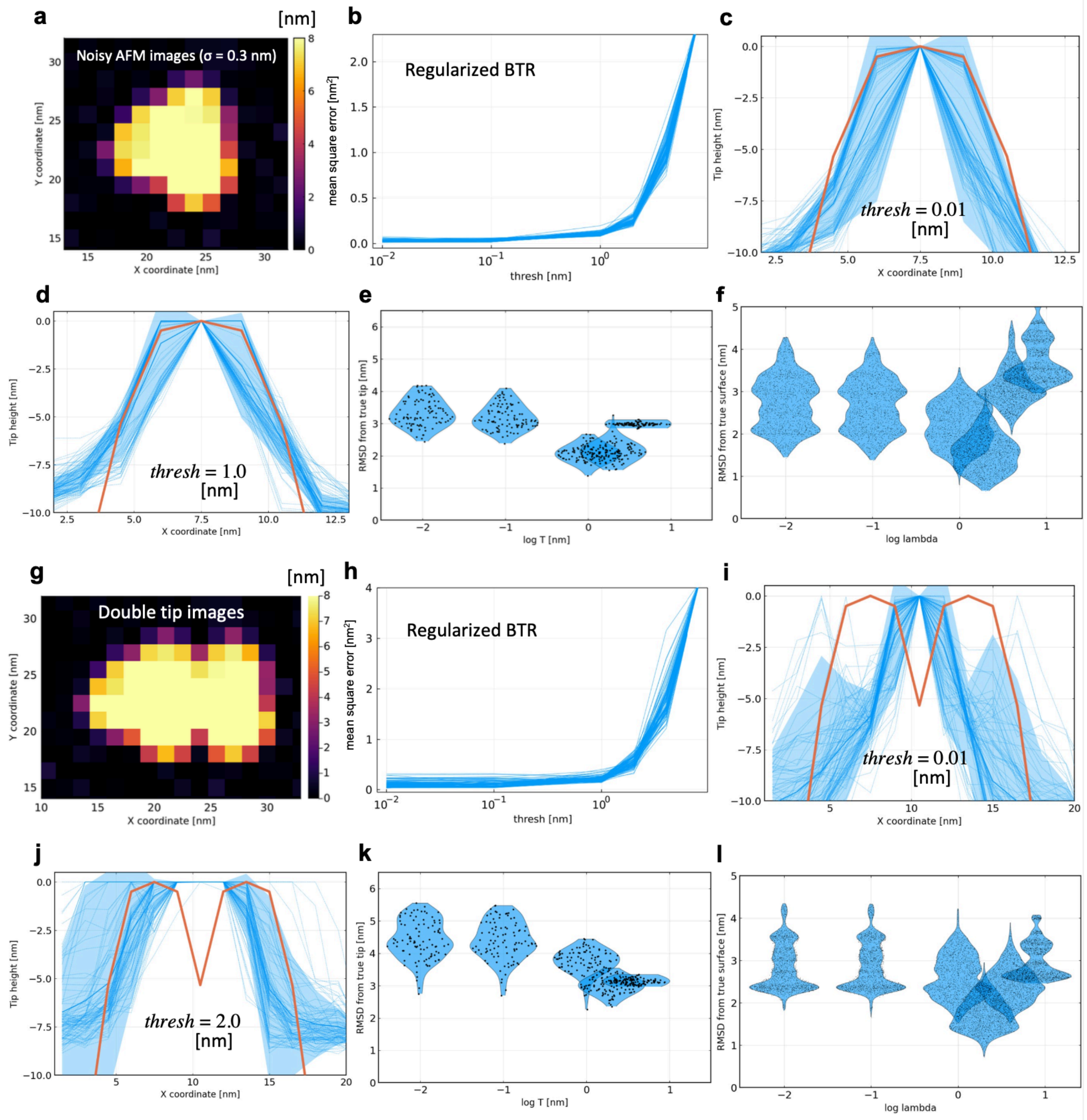

Supplementary Figure 3. Results of twin experiments for the original blind tip reconstruction with a improved regularization scheme. **a**, 1st frame of 20 images used for the twin experiment in noisy conditions. **b**, Mean square errors optimized at various parameter values. **c-d**, Cross sections of reconstructed tip shapes along the x-axis (indicated by dashed blue lines), compared with the ground truth (red line). Shaded area represents the standard deviation. **e**, Root mean square deviations (RMSDs) of the reconstructed tips from the ground truth visualized by violin plots. **f**, Reconstructed molecular surfaces by the deconvolutions with the reconstructed tips. **g-l**, Results of twin experiment in a double-tip case.

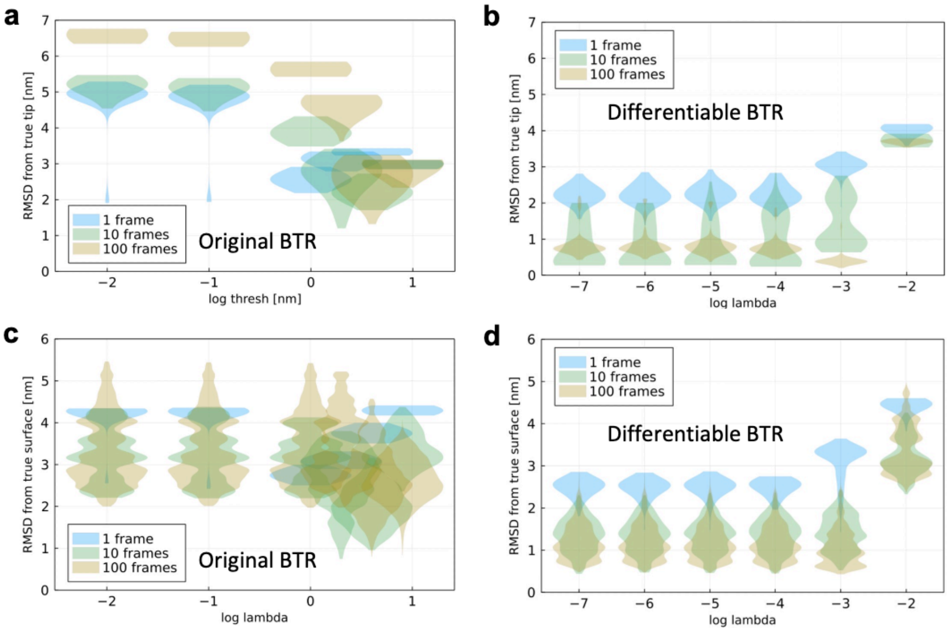

Supplementary Figure 4. Results of twin experiment in single-tip noisy conditions with various number of frames (1 frame, 10 frames, and 100 frames). **a**, Root mean square deviations (RMSDs) of the reconstructed tips with the original blind tip reconstruction (BTR) from the ground truth visualized by violin plots. **b**, RMSDs of the reconstructed tips with the differentiable BTR from the ground truth. **c**, RMSDs of the reconstructed molecular surface with the original BTR from the ground truth. **d**, RMSDs of the reconstructed molecular surface with the differentiable BTR from the ground truth.

Supplementary Movie 1. Reconstructed molecular surfaces of myosin V and actin filament by the deconvolutions with the tips of blind tip reconstructions. The whole frames (30 frames) are converted to a movie of 5 fps.
